# Chandipura Virus: An Emerging Neurological Threat Transmitted by Sandflies in India

**DOI:** 10.1002/iid3.70389

**Published:** 2026-07-14

**Authors:** Abrar Ahmad Zargar, Reena Gupta, P. Krishnamoorthy

**Affiliations:** ^1^ ISF College of Pharmacy Moga Punjab India; ^2^ GLA University Mathura Uttar Pradesh India; ^3^ Centre for Environmental Research, Kongu Engineering College Erode Tamil Nadu India

**Keywords:** acute encephalitis syndrome, Chandipura virus, One Health, Rhabdoviridae, sandfly, vector control, Vesiculovirus, virology

## Abstract

**Background:**

Chandipura virus (CHPV), a neurotropic, negative‐sense RNA virus belonging to the genus *Vesiculovirus* (family *Rhabdoviridae*), has emerged as a significant cause of acute encephalitis syndrome (AES) in India, predominantly affecting children. Since the major outbreaks in 2003–2004 and the recent resurgence in 2024, CHPV has re‐emerged as a high‐fatality, climate‐sensitive arboviral threat transmitted primarily by phlebotomine sandflies. The absence of specific antivirals or licensed vaccines underscores the need for comprehensive understanding and preparedness.

**Methods:**

This narrative review synthesizes available literature on CHPV epidemiology, outbreak history, molecular virology, transmission ecology, neuropathogenesis, host immune responses, diagnostic modalities, therapeutic candidates, and preventive strategies. Data from published outbreak reports, genomic analyses, experimental studies, and public health updates were critically examined to provide an integrated One Health perspective.

**Results:**

CHPV outbreaks have been characterized by rapid disease progression, high case‐fatality rates (often 30%–70%), and marked pediatric predominance. Molecular studies reveal a conserved vesiculovirus genomic organization (3′‐N‐P‐M‐G‐L‐5′), with emerging phylogenomic evidence indicating geographic clustering but limited global genomic representation. Neurotropism is mediated by rapid viral replication in the central nervous system, triggering microglial activation, oxidative stress, cytokine dysregulation, complement interactions, and Fas‐mediated neuronal apoptosis. Diagnosis relies primarily on RT‐PCR during the early viremic phase and IgM ELISA in later stages, though rapid field‐deployable diagnostics remain unavailable. Experimental antiviral strategies, including ribavirin, favipiravir, lycorine, RNA interference, and immunomodulatory approaches, demonstrate preclinical promise but lack human clinical validation. Vector control and environmental management remain the cornerstone of prevention.

**Conclusion:**

Chandipura virus represents a re‐emerging neuroinvasive arbovirus with significant public health implications in India. Strengthening AES surveillance, integrating genomic monitoring, developing rapid diagnostics, advancing antiviral and vaccine research, and implementing climate‐informed vector control strategies are critical priorities. A coordinated One Health framework is essential to mitigate future outbreaks and reduce pediatric mortality.

## Graphical abstract

Chandipura virus (CHPV), a neurotropic arbovirus transmitted primarily by sandflies, has emerged as a significant public health concern in India following recurrent outbreaks with high pediatric fatality rates. This review highlights the virus's virology, transmission dynamics, clinical features, and the urgent need for improved diagnostics, surveillance, and preventive measures, including vaccine development. A One Health approach is emphasized to effectively mitigate the threat posed by this rapidly progressing encephalitic virus.

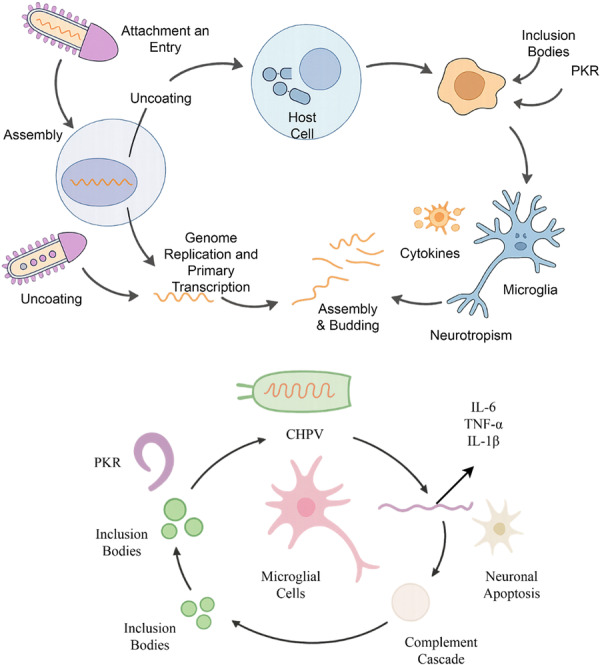

**Abbreviations**
ABC: airway, breathing, circulation
AES: acute encephalitis syndrome
Ag‐RDT: Antigen Rapid Diagnostic Test
BBB: blood–brain barrier
BPL: beta‐propiolactone
BSL‐3: biosafety level 3
CHPV: Chandipura virus
CNS: central nervous system
COX‐2: cyclooxygenase‐2
CypA: Cyclophilin‐A
DNA: deoxyribonucleic acid
ELISA: enzyme‐linked immunosorbent assay
GISAID: global initiative on sharing all influenza data
ICMR: Indian Council of Medical Research
ICU: intensive care unit
IgG: Immunoglobulin G
IgM: Immunoglobulin M
iNOS: inducible nitric oxide synthase
L: large protein (RNA polymerase)
LRP1: lipoprotein receptor–related protein 1
M: matrix protein
miR‐21‐5p: MicroRNA‐21‐5p
MN‐ELISA: micro‐neutralization enzyme‐linked immunosorbent assay
N: nucleocapsid protein
NF‐κB: nuclear factor kappa‐B
NGS: next‐generation sequencing
NNRTIs: non‐nucleoside reverse transcriptase inhibitors
NO: nitric oxide
NRTIs: nucleoside reverse transcriptase inhibitors
P: phosphoprotein
PCR: polymerase chain reaction
PFU: plaque‐forming unit
PI3K/AKT: phosphatidylinositol‐3‐kinase/protein kinase B pathway
PKR: protein kinase R
PRNT: Plaque Reduction Neutralization Test
RNA: ribonucleic acid
ROS: reactive oxygen species
RT‐PCR: reverse transcriptase polymerase chain reaction
SG: stress granule
SI: selectivity index
TIA‐1: T‐cell intracellular antigen‐1
TNF‐α: tumor necrosis factor‐alpha
T‐705: favipiravir
VSV: vesicular stomatitis virus
WHO: World Health Organization

## 1 Introduction

Chandipura virus (CHPV) is a neurotropic, negative‐sense RNA virus of the genus *Vesiculovirus* (family *Rhabdoviridae*) that has periodically appeared as a cause of acute encephalitis syndrome (AES) in India, mainly among children under 15 years of age [1]. First identified in 1965 during a dengue‐like fever outbreak in a town of Maharashtra, CHPV generally remained under‐recognized until the early 2000s, when pediatric encephalitis outbreaks—with exceptionally high case‐fatality rates—attracted attention to its devastating potential [2]. Transmission of CHPV is largely vector‐borne: phlebotomine sandflies (particularly genera *Phlebotomus* and *Sergentomyia*) have been repeatedly implicated, supported by entomological surveys in endemic places (e.g., central India) and experimental vector‐competence studies [3]. Sandfly proliferation is influenced by ecological and climatic factors, including monsoon rainfall, rural dwelling conditions, and environmental sanitation, making CHPV a climate‐sensitive, One‐Health‐relevant arbovirus [4]. The initial febrile illness, neurologic involvement (seizures, altered sensorium, or coma), and, in extreme cases, mortality within 24–72 h of hospitalization characterize the clinical course of CHPV infection [5]. During previous outbreaks, pediatric populations—particularly in rural or resource‐limited settings—have suffered the heaviest burden, underscoring problems of social vulnerability, limited access to crucial care, and high mortality [6]. In 2024, India witnessed the greatest CHPV outbreak in almost two decades. Clusters of AES in children have been recorded in several states, including Gujarat, Rajasthan, Madhya Pradesh, and others; several of these clusters have been verified as CHPV by RT‐PCR or serology; this revival highlights the widening geographic footprint, changing vector ecology, and increased public health risk [6]. Beyond epidemiology and epidemic dynamics, new advancements have expanded understanding of CHPV's biology. The results of a 2025 phylogenomic analysis of 23 publicly accessible CHPV genomes (from humans, sandflies, and other hosts) indicated host‐specific and geographic differences, as well as distinct evolutionary lineages. This finding highlights the necessity of ongoing genomic surveillance to monitor virus evolution and possible spillover events [7]. Molecular detection (RT‐PCR) or serological assays are the mainstays of current diagnostic efforts; nevertheless, accessibility issues, particularly in rural and resource‐poor settings, impede timely detection, leading to delayed diagnosis and worse results [8]. Crucially, no particular antiviral medication or approved vaccination available for CHPV; management remains supportive and often requires intensive care [2]. CHPV is a priority emergent neurotropic virus in India and the larger South Asian region due to this gap, high mortality, rapid disease progression, pediatric sensitivity, and climatic/vector‐driven resurgence. A thorough synthesis is desperately needed in light of the mounting evidence, which includes frequent high‐fatality epidemics, changing ecological and vector dynamics, gaps in diagnosis and treatment, and insufficient genetic surveillance. With a One‐Health perspective to guide future research, surveillance, control, and preparedness strategies, this review attempts to provide an integrated, current framework covering CHPV's molecular virology, transmission ecology and vector biology, clinical‐epidemiology, neuropathogenesis, diagnostic and therapeutic challenges, and translational outlook. The key knowledge gaps and priority future directions for Chandipura virus (CHPV) are given in Table 1.

**TABLE 1:** Key knowledge gaps and priority future directions for Chandipura virus (CHPV).

Domain	Current limitations	Priority future directions	References
Epidemiology & surveillance	Outbreaks detected only after symptomatic pediatric cases surge; lack of routine surveillance	Integrate CHPV testing into national AES surveillance; develop real‐time outbreak tracking	[6]
Vector ecology	Sandfly distribution data incomplete; limited environmental monitoring	Climate‐informed vector mapping; community‐based vector control	[4]
Transmission dynamics	Possible secondary vectors? Limited urban data	Broaden entomological studies (*Aedes*/*Culex* potential); One‐Health ecological surveillance	[3]
Neuropathogenesis	Incomplete mapping of host immune triggers; limited pediatric CNS data	Advanced models to study microglial activation, neuroinflammation, BBB disruption	[5]
Genomic evolution	Few whole‐genome sequences; low sampling density	Continuous genomic surveillance; phylogeographic spread analysis	[7]
Diagnostics	No rapid bedside assays; limited RT‐PCR access in rural hospitals	Develop point‐of‐care molecular/antigen tests; multiplex arbovirus panels	[8]
Therapeutics	No approved antivirals; ribavirin/favipiravir efficacy unproven in humans	Conduct clinical evaluation of antiviral candidates; explore immunomodulation	[2]
Vaccines	Only inactivated and subunit candidates tested in laboratory/animal settings	Advance CHPV vaccines to Phase I/II human trials	[2]
Public health preparedness	Limited healthcare provider awareness; outbreak response inconsistent	AES education, rural ICU strengthening, pediatric emergency readiness	[5]

### 1.1 Epidemiology and Outbreak

Since the early 2000s, India has experienced sporadic but severe outbreaks of acute encephalitis syndrome (AES) caused by the Chandipura virus (CHPV), with a constant prevalence in children. It is vital to distinguish between AES surveillance counts and laboratory‐confirmed CHPV cases, as only a portion of AES episodes are etiologically associated to CHPV [5, 9, 10, 11, 12]. When 329 AES cases and 183 deaths (CFR = 55.6%) were reported in children in Andhra Pradesh in 2003, it was the first significant incident of CHPV‐associated encephalitis. About half of the examined AES cases had CHPV RNA or antibodies, according to later virological investigations, proving that the virus is a significant etiologic factor but not the only reason for all AES presentations [12]. Shortly later, a 2004 outbreak in eastern Gujarat featured 26 probable encephalitis cases in children with a CFR of 78.3%; CHPV was isolated from both human samples and sandflies, confirming a focal but highly fatal pandemic [13]. In addition, rare pediatric deaths and small clusters were recorded from Gujarat in 2014 and 2016, and isolated fatal cases continued to arise, showing low‐level endemic circulation with periodic focal amplification [14]. In 2024, there was the biggest documented revival in more than 20 years, mostly in Gujarat but also in Madhya Pradesh and Rajasthan. The Ministry of Health and Family Welfare (MoHFW) of India reports that as of July 31, 2024, there were 148 documented AES cases (140 from Gujarat and 8 from neighboring states) with 59 deaths, 51 of which were youngsters with laboratory‐confirmed CHPV infection [15]. There were 245 AES cases and 82 deaths (CFR 33%), including 64 laboratory‐confirmed CHPV infections, according to a later WHO Disease Outbreak News update that reported data until mid‐August. This update emphasized that only a percentage of AES cases were virologically verified as CHPV [6]. Independent outbreak assessments and media‐verified public‐health reports describe 133–148 AES cases and 47–51 confirmed CHPV infections in Gujarat alone, with most patients being youngsters under 15 years [16]. Pediatric age groups have consistently accounted for the majority of severe and fatal cases in outbreaks since 2003; reported case‐fatality rates for confirmed or strongly suspected CHPV encephalitis typically range from approximately 30% to over 70%, depending on the outbreak, case definition, and denominator employed [5]. However, insufficient testing of AES cases, regional and temporal variations in diagnostic techniques, and a lack of age‐stratified and laboratory‐confirmed surveillance data continue to make precise burden estimation difficult. Therefore, in this review, we offer AES numbers and CHPV‐confirmed cases separately, with explicit denominators, to avoid overestimation of CHPV disease burden while still underlining its high fatality and significant pediatric predilection. The major documented Chandipura virus outbreaks in India with distinction between AES surveillance cases and laboratory‐confirmed CHPV infections are given in Table 2.

**TABLE 2:** Major documented Chandipura virus outbreaks in India with distinction between AES surveillance cases and laboratory‐confirmed CHPV infections.

Year	Region	Surveillance denominator	AES/suspected cases	Laboratory‐confirmed CHPV cases	Deaths (*n*)	Approx. CFR (%)	Predominant age group	References
2003	Andhra Pradesh (multiple districts)	AES surveillance	329 AES	~50%–60% of tested AES positive for CHPV (RT‐PCR/serology)	183	≈55.6 (AES)	Children (mostly < 15 years)	[17]
2004	Gujarat (Vadodara & Panchmahal districts)	Outbreak investigation (probable encephalitis)	26 probable encephalitis	CHPV isolated from patients & sandflies (number of positives not equal to all 26)	18–20 (CFR ≈ 78%)	≈78 (probable cases)	Children	[13]
2009	Maharashtra	Confirmed CHPV cases	– (not systematically reported as AES)	52 confirmed CHPV	15	≈28.8 (confirmed)	Mostly children	[18]
2010	Gujarat (Kheda, Vadodara, Panchmahal)	Confirmed CHPV cases		50 confirmed CHPV	16	32 (confirmed)	Mostly children < 14 years	[18]
2009–2011 (total)	Maharashtra & Gujarat	Confirmed CHPV cases		110 confirmed CHPV	31	28–30 (confirmed)	Children	[18]
2014, 2016, 2019	Gujarat (sporadic)	Single cases/small clusters	Isolated AES/encephalitis cases	Individual CHPV‐positive cases	1 each reported year	Not applicable (case series)	Pediatric	[19]
2024	Gujarat + Rajasthan + MP + Maharashtra	AES surveillance + lab confirmation	148–245 AES (depending on cut‐off date and reporting source)	51–64 confirmed CHPV	59–82 (AES deaths)	≈33% (AES); higher among confirmed cases	Children < 15 years	[20]

### 1.2 Comparative Outbreak Metrics and Sources of Heterogeneity

The Chandipura outbreaks' reported case‐fatality rates (CFRs) vary greatly in time and location; they ranged from approximately 56% to 75% in the large pediatric outbreaks in Andhra Pradesh (2003) and Gujarat (2004), 43.6% in Nagpur division (2007), approximately 33% for the India‐wide AES cluster in June–August 2024, and 47.36% among laboratory‐confirmed CHPV in the comprehensive Gujarat 2024 investigation. Denominator effects (AES vs. lab‐confirmed CHPV), ascertainment bias during surge periods, variable PICU access and time‐to‐admission, and developing diagnostics (RT‐PCR adoption since 2008) and triage processes are all factors that contribute to these discrepancies. Therefore, it is important to exercise caution when interpreting cross‐outbreak CFR comparisons, giving preference to lab‐confirmed series with defined case definitions [17]. Although modern series integrate RT‐PCR confirmation with expanded AES screening, earlier reports often employed AES as the surveillance denominator with little virologic confirmation. This results in lower apparent CFRs by catching milder or non‐CHPV etiologies in the denominator. The availability of standardized inactivated‐antigen ELISAs and the development of a validated real‐time one‐step RT‐PCR assay have increased trend analysis specificity [21].

### 1.3 Molecular Virology, Viral Proteins and Genomic Diversity of CHPV

**FIGURE 1:**
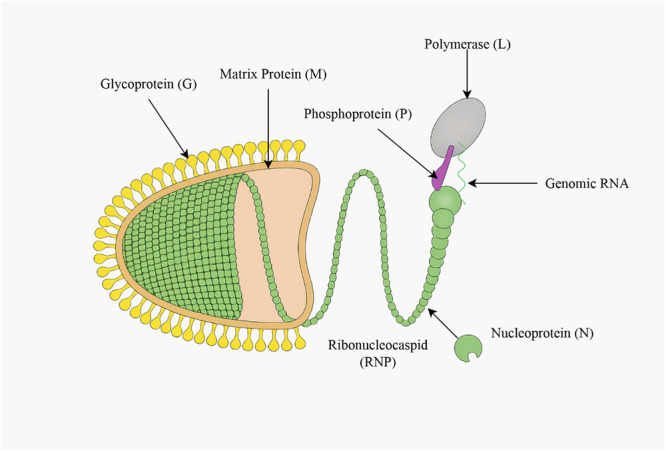
Schematic representation of Chandipura virus (CHPV) structure.

The Chandipura virus (CHPV) belongs to the genus *Vesiculovirus* (family *Rhabdoviridae*) and is a bullet‐shaped, enveloped, negative‐sense RNA virus. Its ~11.1 kb genome follows the classic vesiculovirus organization 3′‐N‐P‐M‐G‐L‐5′ and encodes five key proteins: the nucleoprotein (N), phosphoprotein (P), matrix protein (M), glycoprotein (G), and the large RNA‐dependent RNA polymerase (L) [2]. The ribonucleoprotein (RNP) complex is formed when N encapsulates the genomic RNA, whereas P functions as a non‐catalytic cofactor of L and forms the viral transcriptase/replicase with L. While G is a type I transmembrane glycoprotein that facilitates receptor binding and pH‐dependent fusion in endosomes, M connects the RNP to the viral envelope and aids in assembly and budding [22]. The prototype vesiculovirus vesicular stomatitis virus (VSV), which shares a conserved genomic layout and overall virion architecture, provides most of the functional understanding of CHPV. Direct experimental data on individual CHPV proteins are still few [22]. M is the primary driver of virion assembly for VSV, according to structural and biochemical research. It oligomerizes, connects the nucleocapsid to the inner leaflet of the plasma membrane, and encourages budding. It also aids in host shut‐off by preventing mRNA nuclear export and transcription [23]. By homology, CHPV M is anticipated to serve similar activities, although this assumption is based mostly on sequence conservation of late (L‐) domain and membrane‐interacting motifs rather than direct mechanistic investigations and hence must be taken cautiously [24]. Similar to VSV G, CHPV G is thought to mediate viral attachment and low‐pH‐triggered fusion in endosomes; however, specific receptor use and cell‐type‐specific entry mechanisms for CHPV are still unclear [2]. Which features of CHPV biology are supported by virus‐specific data has started to become clear through comparative genomics. Indian isolates from the 1965 prototype and the 2003–2007 outbreaks were subjected to whole‐genome sequencing, which revealed that while CHPV is still a typical vesiculovirus at the genome level, it has accumulated amino acid changes in functionally significant areas. Notably, Cherian et al. identified sequence variation in the L‐domain surrounding area of M, which in VSV is critical for contacting host budding machinery, and mapped numerous mutations in G to predicted antigenic locations, as well as a mutation in N within a suspected T‐cell epitope [24]. Intrinsically disordered regions spanning CHPV proteins may be involved in host‐protein interactions and immune evasion, according to a more recent “dark proteome” analysis, which emphasizes the necessity of focused functional research once more [25]. In comparison to other neurotropic RNA viruses, CHPV is still significantly under‐sequenced despite these developments. A 2025 phylogenetic research evaluated only 23 publicly accessible whole genomes—derived from human cases, sandflies and a single hedgehog isolate—and found indications of host‐ and geography‐associated clumping within this constrained dataset [7]. The scientists did, however, specifically note that strong inference regarding global evolutionary dynamics, transmission channels, or the formation of stable lineages is limited by the small sample size, high overrepresentation of Indian sequences, and sparse temporal coverage [7]. Similarly, sequencing of viruses from the massive 2024 Gujarat outbreak demonstrated that modern clinical isolates are substantially comparable to the historical 1965 prototype strain, with just modest nucleotide mutations and no major variations in anticipated protein structure or antigenic areas [26]. There are two significant ramifications to these observations. First, mechanistic conclusions about CHPV proteins should be clearly distinguished into those supported by CHPV‐specific experimental data (e.g., sequence variation in M, G, and N; predicted disorder and interaction motifs) and those inferred from the broader vesiculovirus literature (e.g., detailed roles of M and G characterized in VSV). Second, the interpretation of existing phylogenies must be conservative: current whole‐genome datasets are too scarce and skewed to make strong assertions about CHPV clade structure, evolutionary rate shifts or adaptability [2]. Instead, the main and strongest finding from the research that is currently available is the urgent need for more extensive and geographically representative sequencing, including routine whole‐genome characterization of outbreak isolates and strains derived from vectors, in order to enable accurate molecular epidemiology, identify possible emergence of antigenically distinct variants, and guide the design of vaccines or antivirals [27]. The schematic representation of Chandipura virus (CHPV) structure is given in Figure 1.

### 1.4 Life Cycle of Chandipura Virus

Five structural proteins—nucleocapsid (N), phosphoprotein (P), matrix (M), glycoprotein (G), and large polymerase (L)—are encoded by the negative‐sense, single‐stranded RNA genome of the enveloped, bullet‐shaped Chandipura virus (CHPV), a member of the *Rhabdoviridae* family. The functional core that propels transcription and replication in the cytoplasm is the ribonucleoprotein (RNP) complex, which is made up of genomic RNA that is firmly encapsulated by N and linked to the L–P RNA‐dependent RNA polymerase [28].

### 1.5 Attachment and Entry

When the G glycoprotein on the viral envelope attaches itself to particular molecules on the surface of the host cell, CHPV causes infection. According to recent proteomic and biochemical research, α2‐macroglobulin (A2M) and its receptor low‐density lipoprotein receptor–related protein‐1 (LRP1), as well as GRP78, are important host factors that interact with CHPV and modulate infectivity, suggesting an A2M–LRP1 axis in viral attachment and entry [29]. Additionally, it has been demonstrated that cell‐surface vimentin functions as an attachment factor that collaborates with G protein to support adsorption, internalization, and effective viral generation while also affecting antibody recognition [30]. CHPV usually enters cells through clathrin‐mediated endocytosis after receptor engagement. Acidification of the endosome causes conformational changes in G that promote fusion of the viral envelope with the endosomal membrane, releasing the RNP into the cytoplasm [8].

### 1.6 Uncoating, Primary Transcription and Genome Replication

After entering the cytoplasm, N continues to coat the RNP, protecting the RNA and acting as a template for the L–P polymerase complex. Using a stop‐start mechanism characteristic of rhabdoviruses, the polymerase initially performs “primary transcription” to create a series of capped and polyadenylated viral mRNAs in a gene‐order‐dependent gradient [31]. Viral proteins are produced by host ribosomes from these mRNAs. The polymerase transitions from transcription to replication as N builds up: full‐length antigenomic (+) strands are created, encapsidated by N, and used as templates to create offspring genomic (–) strands, which are then firmly wrapped in N once again to form new RNPs. The crucial significance of intrinsic disorder in N, P, and M proteins is highlighted by structural and bioinformatic investigations. This disorder promotes the dynamic protein–protein interactions necessary for effective transcription–replication cycling and assembly [25].

### 1.7 Assembly, Budding and Release

Newly produced RNPs move to locations at the plasma membrane's inner leaflet that are rich in M and G. Through conserved late (PPSY) motifs and interactions with host factors such CTDNEP1 and ABCE1, the M protein connects the nucleocapsid to the viral envelope and coordinates virion assembly, host mRNA shut‐off, and cytopathic effects [25]. After passing via the ER–Golgi pathway, G glycoprotein molecules are inserted into the plasma membrane, where they group together to create budding sites. At these locations, coordinated recruitment of RNPs and M results in the budding of bullet‐shaped virions, during which the virus releases its lipid envelope from the host membrane to infect nearby cells or spread throughout the body [32].

### 1.8 Inclusion Bodies and Replication Factories

Dense cytoplasmic inclusion bodies (IBs), which function as membraneless replication factories, are formed by CHPV during active replication. According to recent research, these IBs develop via liquid–liquid phase separation and specifically attract the stress‐granule protein TIA‐1 and host protein kinase R (PKR). Viral transcription and virion generation are dramatically reduced when PKR or TIA‐1 are depleted, suggesting that CHPV repurposes stress‐response machinery for proviral activities [33]. Numerous host proteins involved in vesicle trafficking, cytoskeletal organization, and RNA processing engage with CHPV proteins, supporting various stages from entry and uncoating to assembly and egress, according to network‐based interactome analysis [34].

### 1.9 Neurotropism and CNS Life Cycle

Clinical observations and experimental infections in mouse models show that viruses replicate quickly in brain cells, with viral loads peaking in a matter of hours and being closely linked to severe encephalitis in children [28]. In the central nervous system, CHPV infection causes Fas‐mediated neuronal apoptosis and strong microglial activation, which increases neuronal loss beyond directly infected cells and kills uninfected neurons as bystanders [35]. Therefore, the entire life cycle of CHPV in the brain includes both the traditional stages of viral replication and budding, as well as a crucial immune‐mediated stage when disease is largely caused by virus‐driven neuroinflammation and glial responses. The change from direct viral cytopathicity to immunopathogenesis is thus marked by the conclusion of the CNS replication cycle, which starts the neuroimmune cascades described in the following section.

### 1.10 Vector Component of the Life Cycle

**FIGURE 2:**
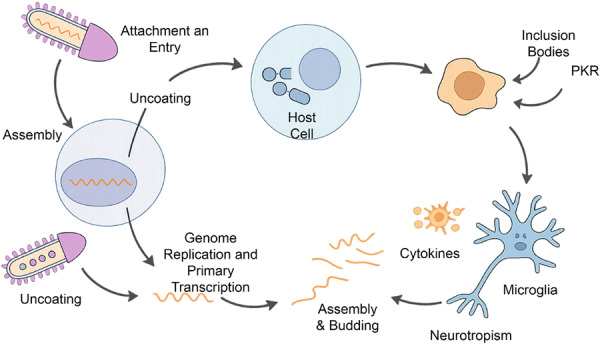
Life cycle and neuroinflammatory pathogenesis of Chandipura virus (CHPV). A schematic representation of the Chandipura virus life cycle showing viral attachment to the host cell, endocytosis, uncoating of the ribonucleoprotein complex, cytoplasmic genome replication, and primary transcription, followed by assembly and budding of progeny virions. Viral replication induces inclusion body formation through ribonucleoprotein interactions, which contribute to immune activation. Neurotropism of CHPV leads to neuronal infection and microglial activation, promoting the release of inflammatory cytokines and contributing to encephalitic pathology.

Both transovarial and venereal transmission have been reported, enabling vertical maintenance within vector populations. In nature, CHPV is mostly maintained in Phlebotomine sandflies, where the virus replicates in the midgut and spreads to the salivary glands [28]. During blood feeding, infected sandflies inject the virus into vertebrate hosts, connecting the previously mentioned mammalian brain life cycle with the in‐vector replication cycle [4, 36, 37]. The life cycle and neuroinflammatory pathogenesis of Chandipura virus (CHPV) are given in Figure 2.

## 2 Host Immune Response and Immunopathogenesis

**FIGURE 3:**
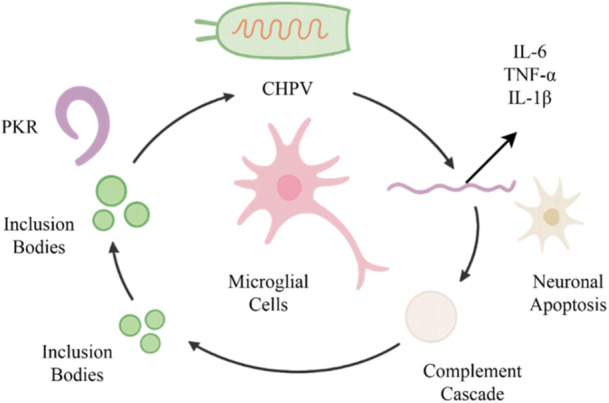
Schematic representation of the host immune and cellular mechanisms involved in Chandipura virus (CHPV) neuropathogenesis.

A central nervous system (CNS) infection with the Chandipura virus (CHPV) causes a strong innate immune response, which directly damages neurons. Experimental murine models have shown that CHPV infection activates microglia in the hippocampus and cortical regions, changing them into an ameboid shape linked to an excess of reactive oxygen species (ROS), cyclooxygenase‐2 (COX‐2), nitric oxide (NO), and inducible nitric oxide synthase (iNOS). Infected brain tissue exhibits widespread neuronal death in correlation with these neuroinflammatory mediators [38]. Verma et al. demonstrated that CHPV‐infected microglia's supernatants were adequate to cause bystander neuronal death in vitro, highlighting the fact that microglial hyperactivation, not direct neuronal infection, is a key factor in CHPV neuropathology [38]. It has been observed that vesiculoviruses, including vesicular stomatitis virus, exhibit similar oxidative and inflammatory cascades, indicating conserved pathogenic mechanisms [39]. In addition to activating microglia, CHPV infection alters host gene networks that control cytokine signaling and compromises the integrity of the blood–brain barrier (BBB) [40]. Pandey et al. showed that infection of human microglial cells activates the PI3K/AKT/NF‐κB axis, down‐regulates PTEN, and up‐regulates miR‐21‐5p, resulting in increased production of IL‐6, TNF‐α, and IL‐1β, which are important mediators of CHPV‐induced neuroinflammation [40]. Although complement activation may aid in the early neutralization of viruses, CHPV shows some resistance to serum complement, maybe as a result of surface glycoprotein shielding [41]. Together, these results present a two‐phase pathogenic model: (i) oxidative stress and neuroinflammation triggered by microglia, and (ii) breakdown of the blood‐brain barrier with complement evasion, which leads to fast neurologic decline, especially in pediatric patients. Nonetheless, thorough mapping of complement‐mediated damage and cytokine/chemokine kinetics during human CHPV encephalitis is still an unfulfilled research goal [33]. The schematic representation of the host immune and cellular mechanisms involved in Chandipura virus (CHPV) neuropathogenesis is given in Figure 3.

### 2.1 Pathogenesis & Host Immune Responses

CHPV is a vector‐borne virus that has no viable host in humans. Canonical stress granules (SGs) are not created during replication; instead, CHPV proteins phase‐separate into cytoplasmic inclusion bodies (IBs), which co‐condense SG proteins (PKR, TIA‐1) and facilitate replication. This process has now been demonstrated to be proviral for CHPV [33]. Current research on CHPV has not definitively shown how it interacts with other host cofactors like Cyclophilin‐A (CypA) or surface vimentin; nevertheless, similar functions have been reported for other negative‐strand RNA viruses and should be investigated [42]. After infecting neurons, CHPV activates caspase‐8/−3 pathways in neuronal cultures, causing Fas/FasL‐mediated apoptosis [43]. Microglial activation in CHPV infection models causes the release of pro‐inflammatory cytokines and chemokines, as well as NF‐κB, which can cause unintended neuronal death in nearby cells [44]. Clinical evidence and mouse models indicate that children are disproportionately impacted, despite the paucity of thorough age‐susceptibility investigations. This is consistent with the increased vulnerability of immature brain systems.

It has been demonstrated that the Chandipura virus reprograms the metabolism of cholesterol in neurons. Apolipoprotein E (ApoE), LDL receptor, SREBF‐1, and NSDHL expression are all up‐regulated in infected mouse brains, which raises the amount of cholesterol in neurons. The accumulation of 24(S)‐hydroxycholesterol, which is neurotoxic and increases Fas‐mediated neuronal death during CHPV infection, is subsequently triggered by cholesterol 24‐hydroxylase (CYP46A1) [45].

Neuronal apoptosis brought on by CHPV has been directly linked to calcium signaling. Research conducted both in vitro and in vivo demonstrates that CHPV infection increases intracellular Ca2+ release induced by angiotensin II, which causes mitochondrial dysfunction, elevated reactive oxygen species (ROS), and p38 MAPK activation. The FasL–FADD pathway and caspase‐mediated neuronal apoptosis are influenced by this Ca2 + –ROS–p38 axis; minocycline therapy reduces Ca2+ increase, ROS generation, and subsequent cell death [46].

In normal human serum, CHPV is extremely sensitive to complement. According to in vitro research, the virus is effectively destroyed by activating the classical complement system in a C1q‐, C3‐, and C4‐dependent manner. Direct binding of C1q to CHPV particles results in the deposition of C3b/C4b and virion aggregation; virus aggregation, as opposed to membrane attack complex‐mediated virolysis, neutralizes the virus [47].

The alternate complement activation route also plays a role in CHPV neutralization in vitro, according to a related study. In a factor‐B‐dependent manner, normal human serum from the majority of donors decreased viral titers and CHPV‐positive cells, highlighting the advantageous impact of the alternative pathway in infection control [48].

While mechanisms such as RIG‐I–dependent viral RNA sensing, NF‐κB‐regulated viral survival, and complement‐driven neurotoxicity have been described in other neurotropic mononegavirales, these pathways remain insufficiently defined for Chandipura virus and require further investigation. Addressing these gaps will be essential to fully elucidate the immunopathogenic cascades that drive the rapid neurological deterioration seen in pediatric CHPV infections.

### 2.2 Transmission of Chandipura Virus

**FIGURE 4:**
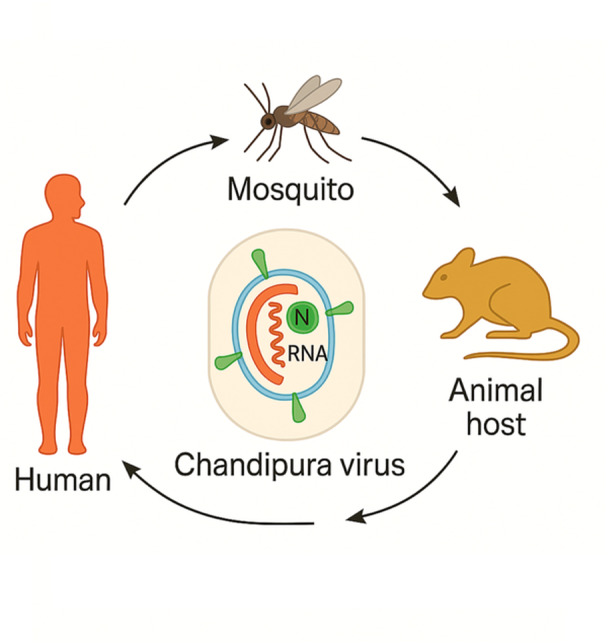
Diagrammatic representation of transmission of Chandipura virus.

It mostly affects children and presents with symptoms resembling influenza and neurologic dysfunctions. Its vectors include sand flies, ticks, and mosquitoes [49, 50]. Both the *Phlebotomus* and *Sergentomyia* genera of sandflies have been linked to the spread of CHPV. For instance, live virus has been recovered from *Sergentomyia* species in endemic locations, while CHPV RNA has been found in *Sergentomyia* pools from Andhra Pradesh (Kolanur) in India. *Phlebotomus argentipes* can infect mice orally and spread CHPV, according to experimental research. *Aedes aegypti* has shown both vertical (transovarial) and venereal (sexual) modes of CHPV spread in laboratory settings in vector competence investigations; filial infection rates of approximately 1.2% and venereal transmission to females of approximately 32.7% have been reported [37, 51, 52, 53]. The isolation of CHPV from *Sergentomyia* sandflies during Maharashtra epidemic investigations and the experimental transmission/vertical or venereal transmission seen in *Phlebotomus* spp. corroborate vector incrimination. Pre‐monsoon sandfly management in endemic districts is justified by these facts [37, 54]. The transmission of Chandipura virus is given in Table 3 and Figure 4.

**TABLE 3:** Transmission of Chandipura virus.

Mode of transmission	Details	References
Vector‐borne transmission		
Sandflies (*Phlebotomus* species)	Sandflies, more especially *Phlebotomus* species, are the main vectors of the Chandipura virus.	[55]
Mosquitoes (*Aedes* and *Culex* species)	Additionally, the virus can be spread via *Aedes aegypti* and *Culex quinquefasciatus* mosquitoes.	[21]
Experimental transmission		
Increased sandfly activity	During the monsoon season, when sandfly numbers are greater, outbreaks frequently take place.	[55]
Preventive measures		
Vector control	Using pesticides, bed nets, and repellents to keep mosquito and sandfly populations under control.	[56]

## 3 Diagnosis

Childhood illnesses progress swiftly and can be fatal in as little as 24–48 h after symptoms appear. This made an early diagnosis necessary. Real‐time and reverse transcriptase polymerase chain reaction (RT‐PCR) are two standardized methods for primary diagnosis [57]. The foundation of diagnosis during the early viromic window (~0–4 days post start) is real‐time one‐step RT‐PCR; its efficacy and quantification for CHPV were initially established [21]. In India, the ICMR‐NIV CHPV IgM ELISA is frequently utilized for field surveillance and post‐acute cases, particularly in the investigations of the 2024 outbreak [58]. Reverse transcription‐based methods are employed to identify viral RNA rather than IgM anti‐CHPV antibodies [49]. G‐protein–based ELISA methods were employed in previous diagnostic studies to detect anti‐CHPV antibodies. Each donor's blood samples were tested for IgG anti‐CHPV antibodies, as was previously indicated [59]. Commercial sources like as Creative Biolabs and BioVenic offer recombinant CHPV glycoprotein (G), mainly for use in immunoprecipitation, Western blot, ELISA, and vaccine development. These goods are clearly marked “for research use only,” and there is no evidence that they have received diagnostic clearance for use in clinical or commercial settings [60]. In academic research, seroconversion after vaccination or infection in animal models has been detected using experimental IgM and neutralizing antibody ELISAs. Nevertheless, they are still being studied and have not been authorized or validated for regular diagnostic use, particularly in clinical settings [61]. Although it is subjective and time‐consuming, the Plaque Reduction Neutralization Test (PRNT) is still the gold standard for neutralizing antibodies. The creation of a micro‐neutralization ELISA (MN‐ELISA) with quicker readouts is described in the article; this test may find application in vaccine trials and Sero‐surveillance [62]. The clinical resemblance of CHPV infections to other AES agents and Japanese encephalitis makes clinical diagnosis more difficult and emphasizes the use of multi‐molecular panels [1]. The comparison of diagnostic assays for Chandipura virus (CHPV) is given in Table 4.

**TABLE 4:** Comparison of diagnostic assays for Chandipura virus (CHPV).

Assay type	Target	Reported sensitivity	Key advantages	Major limitations	References
Real‐time one‐step RT‐PCR	CHPV P gene (TaqMan)	Detection limit: 1.2 × 10⁰ PFU/mL; specificity 100% vs. other viruses	High sensitivity and specificity; rapid turnaround	Requires specialized equipment and trained staff; expensive for field settings	[63]
Nested RT‐PCR/conventional RT‐PCR	CHPV‐N‐gene/N‐gene/another conserved region	Similar detection limit to real‐time in some studies	Broadly accessible, known technique	Greater risk of contamination; slower; lower automation	[57]
IgM/IgG ELISA	Anti‐CHPV antibodies in serum/CSF	Early serology often negative; for example, in outbreak, antibodies more frequent after > 4 days of illness onset	Useful for retrospective serosurveys, exposure studies	Delayed antibody response → less useful for acute diagnosis; cross‐reactivity possible	[11]
Plaque Reduction Neutralization Test (PRNT)	Neutralizing antibodies	Considered “gold standard” for neutralizing antibody detection	Highly specific; indicates functional neutralization	Labor‐intensive, requires BSL‐3/animal work; slow turnaround	[62]
Micro‐neutralization ELISA (MN‐ELISA)	Optical‐density readout of neutralizing antibodies	Recently developed for CHPV; faster than PRNT	Faster, more field‐friendly than PRNT; better throughput	Still less widely validated; may require standardization	[62]

### 3.1 Treatment and Antiviral Strategies for Chandipura Virus

There is currently no approved CHPV‐specific antiviral or vaccine, and clinical care is mostly supportive, concentrating on treating secondary infections, managing elevated intracranial pressure, controlling seizures, and stabilizing breathing, circulation, and airways [28]. A broad‐spectrum nucleoside analog called ribavirin has been tested in vitro against CHPV and is used against a number of RNA viruses. Ribavirin suppressed CHPV reproduction in Vero cell plaque‐reduction experiments with an IC²¹ of ~90 μM, especially when administered just before infection or within 1 h after infection. This suggests that ribavirin's antiviral impact occurs early in the replication cycle [64]. In vitro inhibition of CHPV replication has been demonstrated more recently by lycorine, an alkaloid derived from plants with proven broad‐spectrum antiviral action. In several cell lines, lycorine reduced viral titers by more than 2.5 log⁻¹ with no cytotoxicity. According to time‐of‐addition studies, lycorine activates at an early post‐entry stage. Using a homology model of the CHPV L (RNA‐dependent RNA polymerase) protein, docking and free‐energy calculations revealed high‐affinity lycorine‐binding pockets in the RdRp catalytic domain, indicating that lycorine directly targets the viral polymerase and obstructs the synthesis of positive‐strand RNA [65]. As an experimental antiviral approach, small interfering RNAs (siRNAs) targeting CHPV structural genes have been created. In neural cell models, siRNAs that target the matrix (M) and phosphoprotein (P) genes successfully inhibited CHPV replication; methods based on lipid nanoparticles are being studied to carry these siRNAs to the central nervous system [28]. CHPV infection up‐regulates miR‐155, which suppresses Suppressor of Cytokine Signaling 1 (SOCS1), resulting in increased STAT1 phosphorylation, IFN‐β production, and up‐regulation of IFN‐stimulated genes (ISG54 and ISG56), according to recent mechanistic study in human microglial cells. This antiviral interferon response is strengthened when miR‐155 is overexpressed, while viral replication is increased when miR‐155 is decreased [66]. More generally, miR‐155 plays context‐dependent functions in viral infections, altering interferon pathways and inflammatory responses to operate as proviral in some situations and antiviral in others [67]. For instance, FDA‐approved medicines and other compounds have been docked against the nucleocapsid (N) protein to find candidates with favorable binding energies. This is an example of how structure‐based virtual screening has been applied to CHPV. This study proposed a number of possible binders (such as cyclodextrin derivatives and other small compounds) as putative N‐protein‐targeting antivirals and urged more experimental testing for them [68]. Similar in silico repurposing pipelines have been extensively employed for other viruses, such as the dengue virus and SARS‐CoV‐2, where docking results produce extensive lists of “hits,” but only a tiny percentage eventually advance to in vitro and in vivo efficacy [69]. Although these findings show that CHPV is vulnerable to polymerase‐targeting nucleoside analogs, ribavirin's potential therapeutic application is still experimental because it hasn't been studied in controlled clinical trials for CHPV. Although lycorine is still in the preclinical, in vitro stage and there are currently no animal or human efficacy data available, this work serves as an example of a drug–target–mechanism framework. The promising therapeutics and repurposing are given in Table 5.

**TABLE 5:** Promising therapeutics and repurposing.

Therapeutic strategy	Evidence	Description	Reference(s)
Favipiravir (T‐705)	In vitro, favipiravir completely inhibited CHPV replication at 320 µM, showing EC₅₀ ≈ 92.26 µM and a high selectivity index (SI ≈ 51.24). In vivo, mice given 300 mg/kg/day orally survived 100% to day 7 after lethal CHPV infection	Favipiravir (T‐705): In vitro, favipiravir completely inhibited CHPV replication with a high selectivity index (SI ≈ 51); in mice, oral 300 mg/kg/day achieved 100% survival through day 7 post‐infection—supporting prioritization for early phase trials in pediatric encephalitis due to CHPV	[70]
Antiretroviral NRTIs/NNRTIs (repurposing)	Docking and in‐cell assays show AZT, nevirapine, tenofovir, abacavir binding CHPV polymerase (L) and inhibiting replication. Combination approach proposed	Antiretroviral NRTIs/NNRTIs (repurposing): Docking and cell‐based assays indicate abacavir, tenofovir, zidovudine (AZT), and nevirapine bind the L polymerase and inhibit CHPV replication; authors propose combination therapy as a rational design pending in vivo validation	[71]
Vaccines (preclinical/immunoinformatics)	Inactivated Vero‐cell CHPV vaccine induced neutralizing antibodies and protection in mice. Immunoinformatics analyses of the G glycoprotein predict multi‐epitope subunit candidates (still in silico)	Vaccines (preclinical): A beta‐propiolactone–inactivated Vero‐cell CHPV vaccine elicited robust neutralizing responses and protection in mice, supporting a classical platform as a near‐term candidate; immunoinformatic reports targeting the G glycoprotein suggest feasible multi‐epitope subunit designs, though these remain in silico/preclinical	[72]
Botanical/herbal lead discovery (in silico)	A 2025 in silico screening of Onosma bracteata against CHPV N protein found promising binding affinities (e.g., –8.7 kcal/mol) for Pulmonarioside C, Eritrichin, P‐Coumarinic Acid Ester. Also, a recent in silico dual‐targeting study pointed to apigenin (from Glycyrrhiza glabra) against CHPV + Marburg	Botanical/Herbal lead discovery (in silico): While CHPV‐specific randomized data are unavailable, recent in silico screening pipelines for CHPV (L polymerase and G protein interfaces) have been published and can be extended to plant‐derived libraries to prioritize polyphenols/alkaloids for wet‐lab testing—an approach aligned with current CHPV docking literature	[73, 74]

*Note:* These preclinical and in silico results collectively point to a number of lead candidates, including small compounds and herbal scaffolds, that need more in vitro and in vivo testing. However, none have advanced to human testing, therefore the next paragraphs discuss translation issues and approaches.

### 3.2 Preventive Measures

The following are the main strategies for preventing Chandipura virus infection:

- Practicing good hygiene involves routinely washing your hands with soap and water, especially after handling animals or being in possibly polluted areas [75].
- Targeting areas like sandfly breeding places and using insecticide spraying to eradicate the pests that are rapidly dispersing this illness [76].
- Environmental Control: Using pesticides and environmental management techniques to lessen sandfly habitats [77].
- Public health awareness is the process of informing impacted populations about the dangers of the Chandipura virus and how to protect themselves [77].

Fumigation, pesticide spraying at the community level, and the removal of sandfly breeding sites (such as damp cracks and cow dung walls) are crucial. Plastering walls and enhancing trash disposal are examples of structural upgrades that lessen vector habitat [78].

## 4 Future Directions and Research Gaps

Little is known about how CHPV affects or circumvents the host immune system, particularly in young patients. Although research indicates that brain cells undergo substantial apoptosis, little is known about the function of cytokines, interferons, and adaptive immunity [79]. Despite its preclinical and in vitro effectiveness, ribavirin and favipiravir have not progressed to human trials. BPL‐inactivated vaccines and immunoinformatic‐based vaccination candidates are yet in the preclinical or animal testing phases [70, 80, 81]. There are no field‐deployable, CHPV‐specific fast diagnostics available, despite the fact that RT‐PCR and G‐protein‐based ELISA are employed in research. In India, CHPV is not included in conventional AES differential panels, and there are no commercial kits available [61]. The lack of genomic sequences of CHPV from recent outbreaks makes it extremely difficult to monitor the evolution of strains, transmission networks, and potential recombination events. A comparative genomics study, for instance, only provided the whole genomes of four CHPV samples from outbreaks that occurred between 2003 and 2007 [82]. Although high‐resolution spatiotemporal data are limited, there is emerging evidence that CHPV outbreaks are linked to sandfly habitat shifts and climatic events (monsoon and floods) [83, 84]. CHPV is not covered by national vector‐borne illness programs like JE or Dengue, and there are no vaccination campaigns, community training materials, or epidemic predicting models [8, 85]. Even though India's proposed national AES surveillance system intends to triage 20% of negative Level 1–4 cases to next‐generation sequencing (NGS) for uncommon infections, there is still a significant gap in the integration of tiered diagnostics and monitoring [86], it is still not implemented at the district hospital level. Reflex NGS in conjunction with a multiplex antigen rapid test (Ag‐RDT) panel could improve this and lower “unknown cause” rates while speeding up epidemic response [87]. Climate change models suggest that sandfly vectors will become more suitable under warming conditions, and niche modeling studies demonstrate that *Phlebotomus* species will shift both latitudinally and altitudinally [88, 89]. Combining these forecasts with real‐time sandfly population models, as shown for Mediterranean environments, may enable proactive vector control scheduling prior to the seasonal maxima of CHPV [90]. Lastly, CHPV research continues to undervalue data sharing and policy integration. Comparative research between states would be sped up by requiring that CHPV outbreak sequences, minimum clinical metadata, and algorithmic analytic pipelines be made publicly available within days (e.g., through GISAID or ENA). This strategy has shown promise in other viral outbreaks, such as those caused by SARS‐CoV‐2 and dengue, and it is becoming more and more supported in frameworks for genomic medicine [86]. The conflict between antiviral programs and viral co‐appropriation of stress‐granule machinery is shown in CHPV pathobiology. Early in infection, the alternative complement pathway shows signs of opsonophagocytic control by exerting a protective impact both in vitro and in vivo. On the other hand, CHPV co‐opts stress‐granule proteins (TIA‐1 and PKR) into cytoplasmic inclusion bodies, where their presence paradoxically promotes replication; TIA‐1/PKR disruption, whether genetic or pharmacologic, decreases viral production. Other host factors, such Cyclophilin‐A, aid in replication and might be pharmacologically receptive. MicroRNA programs, like as miR‐155, can emphasize post‐transcriptional levers of control by amplifying type‐I IFN signaling through SOCS1 repression [59]. CHPV is neurotropic in both cell and mouse models, causing blood‐brain barrier disruption, microglial NF‐κB activation with cytokine/chemokine release, and Fas/FasL‐mediated neuronal death, all of which contribute to fast juvenile encephalopathy. It is known that age‐dependent susceptibility occurs: adult mice withstand peripheral challenge, however young hosts die until intracerebral inoculation occurs, which is consistent with the clinical pediatric preponderance. These findings support the use of complementary therapies that alter death‐receptor signaling or microglial activation in addition to antiviral medications [91]. By combining genetic and mechanistic viewpoints, our work goes beyond previous assessments that focus on the epidemiologic burden, monsoon seasonality, and geographical endemicity. While we agree with their recommendation for a One Health strategy that integrates ecological, vector, and human surveillance, we also advise routine viral sequencing in vector and nonhuman hosts to identify emergence or adaptation early [1].

## 5 Conclusion

The Chandipura virus (CHPV) represents a significant emerging risk due to its severe clinical manifestations and its mode of transmission via sandflies. Despite being a relatively recent discovery, CHPV has demonstrated its potential for causing widespread encephalitis outbreaks, particularly in regions with favorable conditions for sandfly proliferation. The virus's ability to induce acute, high‐fatality illness in affected individuals underscores the urgent need for heightened surveillance and public health measures. The historical context of CHPV, including its initial identification and subsequent outbreaks, highlights the importance of continuous monitoring and research. The virus's rapid incubation period and severe symptoms necessitate prompt diagnosis and supportive care to mitigate the impact on affected populations. Additionally, effective vector control strategies are critical in preventing the spread of CHPV and minimizing future outbreaks. Given the zoonotic and climate‐sensitive nature of CHPV, adopting a One Health approach is crucial. This involves integrating human health, veterinary science, and environmental monitoring to detect, prevent, and respond to CHPV outbreaks. Sandflies, as environmental vectors, are influenced by ecological factors such as rainfall, temperature, and habitat availability. Therefore, interdisciplinary collaboration across entomologists, clinicians, veterinarians, ecologists, and public health experts is essential to design comprehensive outbreak control strategies. Ongoing research into the virus's epidemiology, transmission dynamics, molecular biology, and potential vaccine development is essential for improving disease management and control. Public health interventions should focus on strengthening surveillance systems, enhancing community awareness, and investing in sustainable vector control measures to reduce the risk of CHPV transmission. In conclusion, the Chandipura virus poses a significant public health threat that requires coordinated, cross‐sectoral efforts to address and manage. Continued vigilance and a One Health–centered investment in preventive strategies is crucial for safeguarding communities from the impact of this emerging infectious disease.

## Author Contributions

Abrar Ahmad Zargar contributed toward the conceptions or design of the manuscript and manuscript writing. Abrar Ahmad Zargar is first and main author of the manuscript writing and collected data of present study.

## Funding

The authors received no specific funding for this work.

## Conflicts of Interest

The authors declare no conflicts of interest.

## Data Availability

Data sharing is not applicable to this article, as no new data were created or analyzed in this study.
